# Improved Biodistribution and Extended Serum Half-Life of a Bacteriophage Endolysin by Albumin Binding Domain Fusion

**DOI:** 10.3389/fmicb.2018.02927

**Published:** 2018-11-27

**Authors:** Johan Seijsing, Anna M. Sobieraj, Nadia Keller, Yang Shen, Annelies S. Zinkernagel, Martin J. Loessner, Mathias Schmelcher

**Affiliations:** ^1^Laboratory of Food Microbiology, Institute of Food, Nutrition and Health, ETH Zürich, Zürich, Switzerland; ^2^Division of Infectious Diseases and Hospital Epidemiology, University Hospital Zürich – University of Zürich, Zürich, Switzerland

**Keywords:** staphylococci, antibiotic alternatives, biodistribution, half-life, endolysin, albumin binding domain (ABD), LysK, bacteriophage

## Abstract

The increasing number of multidrug-resistant bacteria intensifies the need to develop new antimicrobial agents. Endolysins are bacteriophage-derived enzymes that degrade the bacterial cell wall and hold promise as a new class of highly specific and versatile antimicrobials. One major limitation to the therapeutic use of endolysins is their often short serum circulation half-life, mostly due to kidney excretion and lysosomal degradation. One strategy to increase the half-life of protein drugs is fusion to the albumin-binding domain (ABD). By high-affinity binding to serum albumin, ABD creates a complex with large hydrodynamic volume, reducing kidney excretion and lysosomal degradation. The aim of this study was to investigate the *in vitro* antibacterial activity and *in vivo* biodistribution and half-life of an engineered variant of the *Staphylococcus aureus* phage endolysin LysK. The ABD sequence was introduced at different positions within the enzyme, and lytic activity of each variant was determined *in vitro* and *ex vivo* in human serum. Half-life and biodistribution were assessed *in vivo* by intravenous injection of europium-labeled proteins into C57BL/6 wild-type mice. Our data demonstrates that fusion of the endolysin to ABD improves its serum circulation half-life and reduces its deposition in the kidneys *in vivo*. The most active construct reduced *S. aureus* counts in human serum *ex vivo* by 3 logs within 60 min. We conclude that ABD fusions provide an effective strategy to extend the half-life of antibacterial enzymes, supporting their therapeutic potential for treatment of systemic bacterial infections.

## Introduction

The important human pathogen *Staphylococcus aureus* has acquired various antibiotic resistances over the years. Of particular importance are methicillin-resistant (MRSA) and vancomycin-resistant *S. aureus* (VRSA) (Chambers and Deleo, 2010).

In order to impede this development, new antimicrobials with alternative mechanisms of action and a reduced chance of resistance development are needed. Endolysins are bacteriophage-derived enzymes (peptidoglycan hydrolases, PGHs) with the ability to degrade the peptidoglycan of the bacterial cell wall, thereby causing cell death (Schmelcher et al., 2012).

The most important advantages of endolysins as antimicrobials compared to conventional antibiotics include their rapid killing kinetics, reduced risk of bacterial resistance (Spratt, 1994; Schmelcher et al., 2012), and the high specificity for their target bacteria, leaving commensal and possibly beneficial microorganisms unaffected (Schmelcher et al., 2012).

LysK, the endolysin of the staphylococcal phage K, is an example of a well-characterized PGH active against staphylococci (O’Flaherty et al., 2005). The modular structure of LysK (O’Flaherty et al., 2004) consists of a C-terminal SH3b cell wall binding domain (CBD) (Whisstock and James, 1999), and two enzymatically active domains (EADs): an N-terminal cysteine, histidine-dependent amidohydrolase/peptidase (CHAP) domain (Bateman and Rawlings, 2003), and a centrally located amidase-2 (N-acetylmuramoyl L-alanine amidase). It has been shown that LysK maintains its high activity even if lacking the amidase-2 domain (Becker et al., 2009), and the CHAP domain has been shown to have high activity on its own (Horgan et al., 2009).

The strong anti-staphylococcal activity of LysK and its engineered variants has been demonstrated both *in vitro* and *in vivo* (Kokai-Kun et al., 2007; Rashel et al., 2007; Daniel et al., 2010; Fenton et al., 2010; Gu et al., 2011; Jun et al., 2013; Schmelcher et al., 2014). However, despite some encouraging results with this and other endolysins (Haddad Kashani et al., 2017), systemic administration of most protein-based drugs, including endolysins, is currently hampered by their short serum circulation half-life (Loeffler et al., 2003; Walsh et al., 2003). The decline in drug concentration is characterized by alpha and beta phase decay. After intravenous administration, concentrations decline rapidly as the drug is distributed into the tissues and organs (Lobo et al., 2004). Subsequently, the residual concentration decreases due to inactivation by antibodies (Jawa et al., 2013), degradation by proteases, endocytosis by epithelial cells, and kidney excretion (Werle and Bernkop-Schnürch, 2006).

Various approaches may extend the half-life of proteins (Patel and Benfield, 1996; Matthews et al., 2008; Kontermann, 2011). A promising strategy is fusion of the albumin binding domain (ABD) to the protein of interest (Nygren et al., 1991). ABD binds to serum albumin with strong affinity and forms a complex with a large hydrodynamic volume, avoiding glomerular filtration (Jonsson et al., 2008). Moreover, proteins fused to ABD are indirectly recycled by the FcRn (Chaudhury et al., 2003). Human serum albumin (HSA) has a serum circulation half-life of 3 weeks in humans (Doweiko and Nompleggi, 1991), whereas murine serum albumin (MSA) has a half-life of 35 h in mice (Chaudhury et al., 2003). Proteins recruiting albumin through high-affinity ABDs have been reported to show similar half-lives (Seijsing et al., 2014).

In this study, we investigated the bacteriolytic activity, serum circulation half-life and biodistribution of engineered variants of the endolysin LysK, with and without ABD.

## Materials and Methods

### Plasmid and DNA Construct Design

**FIGURE 1:**
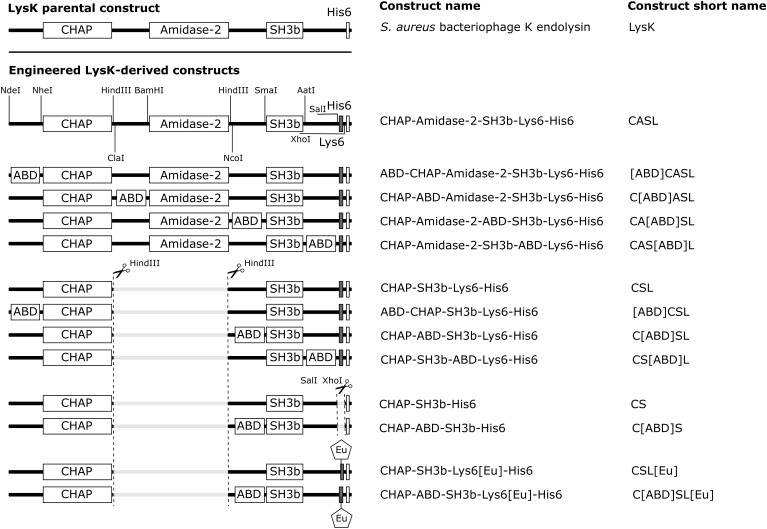
Schematic representation of the DNA constructs created in this study. The DNA constructs contain the genes for the EADs CHAP and amidase-2, the CBD SH3b, a Lys6-tag (gray) for labeling with europium (Eu) via amine coupling, a His6-tag for IMAC purification and the ABD for serum circulation half-life extension. In some constructs, the amidase-2 domain and/or Lys6-tag was removed and the ABD inserted using restriction sites included in the engineered full-length LysK construct CASL. The figure was made using Illustrator for Biological Sequences (IBS) (Liu et al., 2015).

Gene synthesis and subcloning into the pET21a(+) vector (Novagen, Darmstadt, Germany) were performed by BioBasic Inc. (Markham, ON, Canada), and modifications resulting in LysK variants without Amidase-2 domain and Lys6-tag were performed in-house using standard molecular cloning techniques (Green and Sambrook, 2012). Schematics of all created constructs, with the corresponding names and acronyms are presented in Figure 1. The ABD used in this study (ABD035) is an affinity-matured variant (Jonsson et al., 2008) from streptococcal protein G (Nygren et al., 1991; Johansson et al., 2002).

### Recombinant Protein Expression and Purification

Vectors encoding target proteins were introduced into BL21 Gold (DE3) *E. coli* cells (Stratagene, La Jolla, CA, United States) (Table 1) by electroporation. Cells were grown at 37°C and 220 RPM in LB-PE medium (15 g/L Tryptone, 8 g/L Yeast extract, 5 g/L NaCl, pH 7.8) supplemented with 100 μg/mL ampicillin until OD_600_ reached 0.5. Protein expression was induced by adding 0.5 mM IPTG, and cultivation was continued over night at 19°C and 120 RPM. Cells were pelleted using centrifugation and disrupted using both a Stansted Fluid Power Pressure Cell Homogenizer (100 MPa) and sonication on ice/ethanol slurry with a Bandelin Sonopuls HD 2076 (2 min, 1:1 pulses, 50% power). Proteins were purified from cleared crude extracts using nickel affinity chromatography. For the *in vivo* study, the following modifications were made to reduce endotoxin concentrations. Vectors were transformed into electrocompetent *E. coli* ClearColi BL21(DE3) (Lucigen, Middleton, WI, United States) (Table 1). LB-PES (15 g/L Tryptone, 8 g/L yeast extract, 10 g/L NaCl, pH 7.8) medium was used for all cultivations. Bacteria were lysed by sonication only. During purification, the columns were washed twice with 25 ml of ice cold lysis buffer supplemented with 0.1% Triton X-114 (Reichelt et al., 2006), and subsequently 25 ml of lysis buffer. Proteins were tested for endotoxins using the EndoZyme kit (Hyglos, Regensburg, Germany) according to manufacturer’s instructions.

**Table 1:** Bacterial strains used in this study.

Strain	Source	Reference
*Staphylococcus aureus* SA113 (ATCC 35556)	Andreas Peschel, University of Tübingen, Germany	(Kristian et al., 2004)
*S. aureus* Newman D2C (ATCC 25904)	Brigitte Berger-Bächi, University of Zurich, Switzerland	(Bischoff et al., 2004)
*E. coli* BL21-Gold(DE3)	Stratagene, La Jolla, CA, United States	
*E. coli* ClearColi BL21(DE3)	Lucigen, Middleton, WI, United States	

### Europium Labeling

Europium labeling of proteins via the Eu-chelator complex (Sodium[4′-(4′-Amino-4-biphenylyl)-2,2′:6′,2″ -terpyridine-6,6″-diylbis(methyliminodiacetato)]europate(III)) was performed by BioTeZ (Berlin, Germany).

### Surface Plasmon Resonance

For measuring binding of ABD-containing constructs to HSA by surface plasmon resonance (SPR), a Biacore X (GE Healthcare, Uppsala, Sweden) with a C1 sensor chip and HBS-T running buffer was used essentially as previously described (Jonsson et al., 2008).

Human serum albumin (HSA; 100 μg/ml) was immobilized in flow cell 2 using an Amine Coupling Kit (GE Healthcare, Uppsala, Sweden) according to the manufacturer’s instructions. For interaction analysis, 30 μl of endolysin construct at a concentration of 50 nM was injected, with flow cell 1 serving as the reference. The chip surface was regenerated using 15 mM HCl.

### Turbidity Reduction Assay

The assay was performed essentially as described before (Schmelcher et al., 2014), using *S. aureus* SA113 (Kristian et al., 2004) (Table 1) and PBS supplemented with 5 μM HSA (PBS-HSA). Specific enzymatic activity was expressed as ΔOD_600_min^−1^μM^−1^. For comparison of specific activities, one-way ANOVA was performed. The impact of Lys6-tags on activity was assessed by an unpaired *t*-test (GraphPad Prism, 7.02).

### Time Kill Assay

For the time kill assay, *S. aureus* Newman (Bischoff et al., 2004) (Table 1) was grown in LB medium to an OD_600_ of 0.5, and the assay was performed essentially as previously described (Schuch et al., 1986). Target bacteria at a concentration of 10^6^ CFU/ml were mixed with 200 nM of endolysin in human serum (H4522, Sigma-Aldrich, St Louis, MO, United States). Human serum alone was used as negative control. For statistical analysis, two-way ANOVA on log-transformed data followed by Sidak’s multiple comparisons test (GraphPad Prism, 7.02) was performed.

### *In vivo* Half-Life and Biodistribution Study

Eight to ten weeks old female C57BL/6 wild-type mice (Janvier, Le Genest St. Isle, France) were separated into two groups of four mice each. Animals were injected intravenously into the tail vein with ∼7 nmol/kg body weight (100 μl, 1.4 μM) of Eu-labeled C[ABD]SL[Eu] or the control CSL[Eu]. Blood was drawn from the tail vein after 0.25, 24, 72, 120, and 144 h. Heparinized blood was centrifuged and plasma was collected. Mice were euthanized at 144 or 216 h, and organs were homogenized using a TissueLyser (Qiagen, Valencia, CA, United States) in PBS (1:1 weight/volume ratio). The Europium content in the blood and organ samples was determined using an Infinite M1000 PRO (Tecan, Durham, NC, United States) time-resolved fluorescence plate reader. Obtained values were correlated to a spiked standard dilution series of respective proteins in mouse serum or a homogenate of respective organs.

Serum concentrations were plotted against time, and the four last values were used to calculate the beta half-life of the protein using a one phase decay model in Prism (GraphPad Software, San Diego, CA, United States). Student’s *t*-test was used to compare protein concentrations in the serum at the last time point at 144 h. For comparing protein concentrations in organ samples, a two-way ANOVA on log-transformed data followed by Sidak’s multiple comparisons test was performed.

## Results

### Design and Production of Peptidoglycan Hydrolase Constructs

A high affinity variant of ABD [ABD035 (Jonsson et al., 2008)] was selected for fusion to full length and shortened versions of the LysK endolysin. The shortened versions feature a deletion of the centrally located amidase-2 domain, since this domain reportedly contributes little to the overall activity of the enzyme (Becker et al., 2009). Different variants of LysK-ABD fusion constructs were created, with the ABD inserted at various positions within the proteins (Figure 1), in an effort to identify the ABD-tagged version with the highest staphylolytic activity.

All constructs featured a C-terminally located His6-tag to allow purification by immobilized metal ion affinity chromatography (IMAC). In order to allow for directed europium (Eu) labeling, all constructs generated in the first round of cloning contained a Lys6-tag upstream of the His6-tag, thereby reducing the risk of compromising activity by the labeling process. Eu labeling was used to measure the concentration of the proteins in blood and organs using time-resolved fluorescence.

Protein identity and purity of protein preparations were controlled by SDS–PAGE (Supplementary Figure S1A). A prominent additional band of lower than expected molecular weight was observed for all engineered constructs containing the amidase-2 domain, whereas this band was not detected in the parental LysK-His6. Consequently, all constructs yielding the observed truncated protein product were excluded from further experiments.

### High-Affinity Albumin Binding Domain (ABD) Mediates Binding of Peptidoglycan Hydrolase Constructs to Human Serum Albumin

The selected fusion constructs were investigated for their ability to bind to HSA using surface plasmon resonance analysis. HSA was immobilized on the surface of a sensor chip, and interaction of fusion proteins in solution with the immobilized HSA was monitored in real-time. All three protein constructs containing an ABD showed strong binding to HSA, whereas the non-ABD containing control showed weak binding (Supplementary Figure S2). These results suggest that the ABD retains its functionality within the context of the fusion proteins.

### ABD-Containing Constructs Retain Bacteriolytic Activity *in vitro*

**FIGURE 2:**
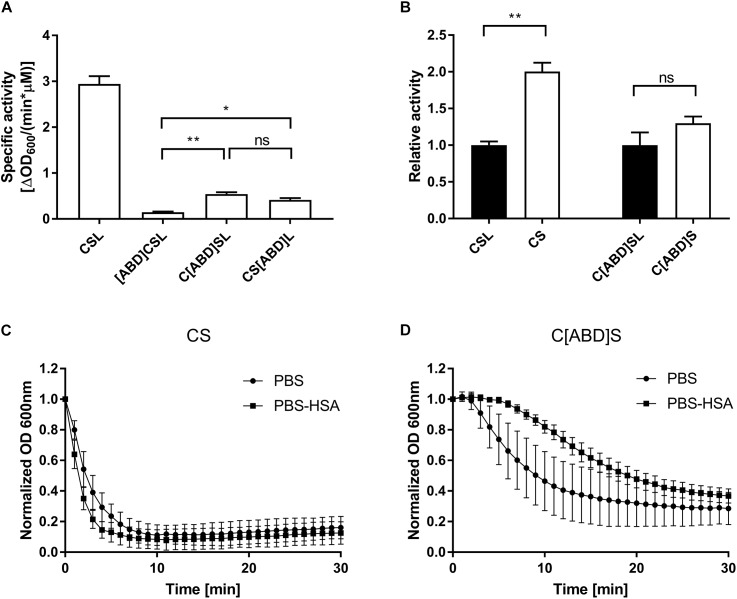
*In vitro* analysis of PGH constructs with and without ABDs by turbidity reduction assays. *Staphylococcus aureus* SA113 substrate cells were mixed with indicated concentrations of endolysin constructs. The reduction in optical density over time was analyzed and the specific activity was calculated as ΔOD_600_/(min^∗^μM). **(A)** Comparison of the specific activities of the control PGH construct CSL and its ABD-containing derivatives. The experiment was performed in PBS-HSA. **(B)** Effect of the removal of Lys6-tags from CSL and its most active ABD-containing derivative. The experiment was performed in PBS-HSA. **(C,D)** Turbidity reduction assays comparing the activity of CS **(C)** and C[ABD]S **(D)** at 125 nM concentration in PBS and PBS-HSA. Data were control-corrected and normalized. Error bars represent the standard error of the mean from 3 **(A,B)** and 4 **(C,D)** individual experiments. ^∗^*p* < 0.05; ^∗∗^*p* < 0.01; ns, non-significant.

The *in vitro* lytic activity of the selected protein constructs was determined by a turbidity reduction assay, which measures the reduction in optical density of a bacterial suspension over time in response to different concentrations of endolysin. The assay was performed in PBS-HSA in order to account for any possible inhibition caused by steric hindrance upon binding of the ABD-containing endolysin constructs to HSA. The most active ABD-containing construct, C[ABD]SL, displayed an activity of 0.54 ΔOD600 min^−1^ μM^−1^ (compared to 2.94 ΔOD600 min^−1^ μM^−1^ for the parental enzyme CSL; Figure 2A) and was selected for further evaluation *in vitro* and *in vivo*.

As a next step, Lys6-tag-free variants of C[ABD]SL and the control CSL were generated, yielding the constructs CHAP-ABD-SH3b-His6 (C[ABD]S) and CHAP-SH3b-His6 (CS), respectively (Figure 1). Lys6-tags had been added to all original constructs to facilitate Eu labeling for *in vivo* experiments. However, they would not be included in enzymes used therapeutically. The Lys6-free constructs showed higher activity in the turbidity reduction assay compared to their tagged counterparts (Figure 2B), for which reason they were chosen for further *in vitro* analysis.

To investigate the influence of HSA on the activity of the control CS and C[ABD]S, both enzymes were compared in turbidity reduction assays performed in PBS only and PBS-HSA. In the presence of HSA, the activity of CS increased slightly as compared to PBS alone (Figure 2C). The reason for this may be that an excess of HSA reduces unspecific binding of the enzyme to the polystyrene of the 96-well plate, increasing the effective concentration of the endolysin construct in the solution. In contrast, the activity of the C[ABD]S construct decreased upon addition of HSA (Figure 2D). It is likely that the HSA binding causes steric hindrance impeding the activity of the endolysin construct or affecting the diffusion rate through the increased size of the complex.

**FIGURE 3:**
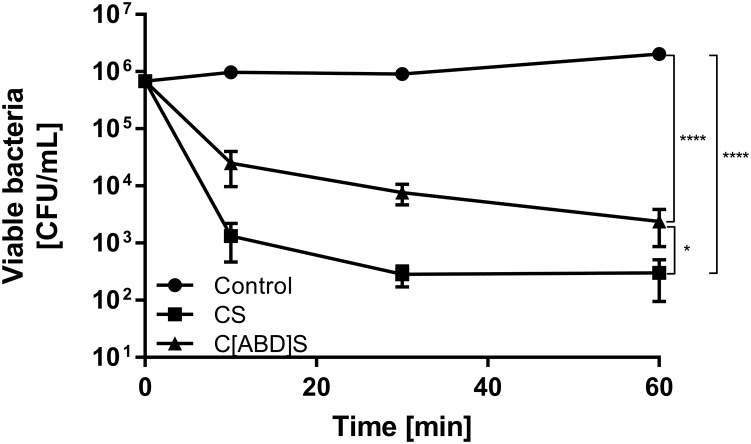
Time kill assay comparing the antibacterial activities of CS and C[ABD]S. *S. aureus* Newman bacteria were mixed with 200 nM of both PGHs in human serum. Serial dilutions of the mixtures were plated at predetermined time points, and colonies were counted after over-night incubation. Data are shown as mean viable bacterial concentrations with error bars representing standard errors of the means from four individual experiments with technical duplicates each. ^∗^*p* < 0.05; ^∗∗∗∗^*p* < 0.0001.

In order to determine the staphylolytic activity of C[ABD]S and CS in an environment mimicking bacteremia, a time-kill assay with the selected constructs in human serum and the clinical isolate *S. aureus* Newman was conducted. Although the ABD-containing construct was less effective than the control enzyme in this experiment, it reduced the number of CFUs by approximately 3 log units within 60 min at a concentration of 200 nM (compared to 4 log units for the control) (Figure 3).

### An Intramolecular ABD Increases the Serum Circulation Half-Life of C[ABD]SL[Eu] in Mice

The Lys6-tagged constructs C[ABD]SL and CSL were selected for investigating the effect of an intramolecular ABD on the serum circulation half-life of these proteins in mice. To this end, preparations of both proteins of high purity (Supplementary Figure S1B) and with endotoxin concentrations <0.05 EU/ml were labeled with the lanthanide europium (Eu) in order to enable measurement of protein concentrations in murine blood and organs by time-resolved fluorescence.

**FIGURE 4:**
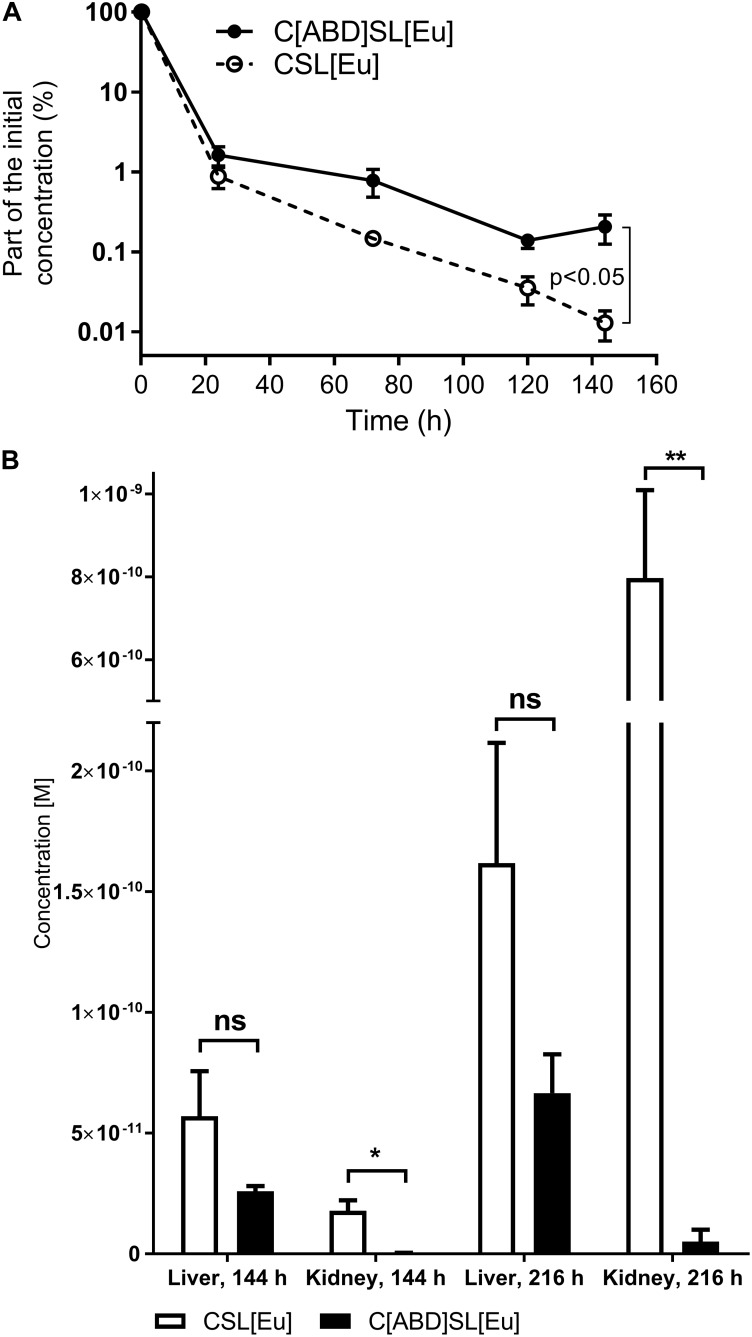
Normalized mean serum concentration-time profiles and biodistribution in murine liver and kidneys of CSL[Eu] and C[ABD]SL[Eu]. C57BL/6 wild-type mice were injected intravenously with the europium (Eu)-labeled protein constructs, blood samples were drawn at predetermined time points, and organs were harvested after euthanasia at 144 and 216 h post-injection. Blood and organs were analyzed for Eu concentration and data are shown as mean concentrations with standard errors of the mean from a total of four mice per group. **(A)** Normalized concentrations of Eu-labeled endolysin constructs in the murine blood at various time points from 0 to 144 h post-injection. **(B)** Eu concentration of the control CSL[Eu] and the ABD-containing C[ABD]SL[Eu] in the liver and kidneys 144 and 216 h post-injection.

The Eu-labeled ABD-containing protein CHAP-ABD-SH3b-Lys6[Eu]-His6 (C[ABD]SL[Eu]) and the control CHAP-SH3b-Lys6[Eu]-His6 (CSL[Eu]) were injected intravenously into C57BL/6 wild-type mice. The blood was sampled at predetermined time points and analyzed for target protein concentrations via time-resolved fluorescence. As previously described for other proteins (Wang et al., 2008; Seijsing et al., 2014), concentrations of both the ABD-containing construct and the control decreased over time following a biphasic process (Figure 4A). During the alpha phase, both concentrations decreased at approximately the same rate (approximately 2 log units within 24 h). However, during the beta phase, the ABD-containing protein showed a slower decrease in concentration than the control. After 144 h, the C[ABD]SL[Eu] concentration in the blood was 16-fold higher than that of the control construct, which was statistically significant (*p* < 0.05). From these data, the serum circulation half-life was calculated to be 23 h for the control CSL[Eu] and 34 h for the ABD-containing C[ABD]SL[Eu].

### ABD Prevents Endolysin Kidney Deposition

In addition to determining the half-life of both constructs in the blood, their biodistribution was investigated by measuring the concentrations of Eu-labeled proteins in various organs of the mice. Mice were euthanized and organs were harvested 144 or 216 h post-injection. Europium was found to accumulate in liver and kidneys over time, with the control CSL[Eu] yielding higher concentrations in both organs and at both time points than the ABD-containing C[ABD]SL[Eu]. This effect was statistically significant in the kidneys and was strongly pronounced at 216 h, where the difference between the constructs was more than 150-fold (Figure 4B). Protein concentrations in the spleen, lung, and heart were below the detection limit for both proteins.

## Discussion

Therapeutic efficacy of a protein-based drug depends on several factors including activity *in vivo*, bioavailability at the site of infection, immunogenicity, and serum circulation half-life. Successful eradication of an infection can be achieved only if the local concentration of the therapeutic antimicrobial agent remains high enough over a sufficient period of time in order to kill the majority of bacteria. In this respect, an extended serum circulation half-life is considered advantageous, since it avoids the necessity of repeated or continuous administration (Walsh et al., 2003; Resch et al., 2011b).

The serum circulation half-lives of PGHs reported in the literature to date are short, ranging from 20.5 min to 1 h (Loeffler et al., 2003; Walsh et al., 2003; Jun et al., 2017). Efforts to extend the half-life of PGHs have been pursued, using PEGylation (Walsh et al., 2003; Resch et al., 2011b) or dimerization (Resch et al., 2011a). By conjugating a polyethylene glycol (PEG) polymer chain to a protein, its hydrodynamic volume increases, which reduces glomerular filtration in the kidneys. In addition, PEGylation can decrease immunogenicity if potential epitopes on the molecule are masked from the immune system (Veronese and Mero, 2008). Unfortunately, the introduction of bulky structures such as PEG has been found to severely affect and even inactivate enzymatic activity of endolysins (Resch et al., 2011b). Moreover, PEGylation is a cumbersome process that may result in multidisperse products, and anti-PEG antibodies have been observed in exposed patients, in addition to non-degraded PEG deposited in patients’ livers (Knop et al., 2010).

An enzyme dimerization strategy has been used with the pneumococcal phage endolysin Cpl-1 (Resch et al., 2011a). In this case, monomeric endolysin molecules were dimerized by introduction of specific cysteine residues, which resulted in a 10-fold decrease in plasma clearance rate. However, this strategy may be successful only for endolysins which exhibit a natural tendency to dimerize, as suggested for Cpl-1 by the authors of the study.

Fusion of endolysins with ABD is a straightforward process that requires no posttranslational chemical modifications. The ABD can be iterated at different positions in the protein until a suitable construct is identified. Clearly, targeted modification represents a major advantage over biochemical approaches as compared to PEGylation, which always result in a heterogeneous mix of products. Not only does the ABD-HSA complex give an increased hydrodynamic volume reducing kidney excretion but, as opposed to PEGylated proteins, it is also rescued from lysosomal degradation through recycling by the neonatal Fc receptor (FcRn) (Chaudhury et al., 2003). Furthermore, recombinant proteins are, unlike PEG, eventually degraded without leaving any chemical traces in the body.

Since endolysins are foreign to the body, they can be immunogenic, as previously reported (Loeffler et al., 2003; Fischetti, 2010; Schmelcher et al., 2012). It can be speculated that the ABD may indirectly dampen such a response. Since ABD fusion proteins form complexes with HSA in the blood, they are recycled back into the blood circulation by the FcRn when endocytosed by epithelial cells or antigen-presenting cells (Chaudhury et al., 2003). Thereby, fusion of ABD to the protein circumvents lysosomal degradation, which would otherwise lead to presentation of immunogenic epitopes by the major histocompatibility complex (MHC) and induce long-lasting B-cell mediated humoral immune response (Bryant and Ploegh, 2004).

In this study, we observed a relatively long half-life of 23 h in mice for the 35 kDa parental enzyme CSL[Eu], which was surprising given the previously published values.

A two- or multi-domain endolysin is likely to show a longer half-life than a globular protein with the same molecular weight due to its larger hydrodynamic volume. However, both Cpl-1 and lysostaphin are also two-domain modular proteins, with half-lives of 20.5 min (Loeffler et al., 2003) and less than 1 h (Walsh et al., 2003), respectively. In addition, the high pI of CSL (9.83) gives the protein a strong positive net charge, possibly resulting in attraction to negatively charged cells and tissues, thus delaying clearance. Such a hypothesis could be sustained considering the low pI of Cpl-1 (Loeffler et al., 2003). However, the pI of lysostaphin is similar, which suggests that the clearance mechanism also depends on other factors (Loeffler et al., 2003; Walsh et al., 2003). The difference in half-life between lysostaphin and the LysK-derivative CSL may also be explained by the different functions of these two enzymes. Lysostaphin is a bacteriocin acting from the outside, therefore relying on efficient diffusion. Thus, it can be reasoned that this protein has been optimized by evolution to minimize interactions with molecules in the environment, which may explain its rapid clearance. In contrast, LysK is a phage endolysin accessing the peptidoglycan from within. Free diffusion through the environment following bacterial lysis would be disadvantageous for the phage, due to the risk of harming not yet infected neighboring hosts (Verbree et al., 2017).

The SAL200 compound is a recombinant version of the staphylococcal endolysin SAL-1 highly similar to LysK. The *in vivo* serum circulation half-life of SAL200 was relatively long when tested in monkeys, ranging from 0.3 to 9.7 h (Jun et al., 2016). However, a recent clinical trial with SAL200 demonstrated a very short serum circulation half-life of only 0.04 to 0.38 h in humans (Jun et al., 2017). This indicates a large discrepancy in the drug’s pharmacokinetics between different organisms, and suggests that the long *in vivo* serum circulation half-life of CSL[Eu] in mice may not be extrapolated to other systems. Here, introduction of an ABD into the CSL protein resulted in a significant increase in serum circulation half-life in mice. The protein construct containing the high-affinity ABD035 showed a half-life of 34 h, which is in line with what has been reported for other ABD fusion proteins previously (Chaudhury et al., 2003; Orlova et al., 2013; Seijsing et al., 2014).

From the biodistribution data, it is evident that the ABD-containing construct C[ABD]SL[Eu] is superior to the non-ABD-containing control in avoiding kidney deposition. As discussed above, this is likely an effect of the differences in hydrodynamic volume and to a smaller extent FcRn recycling (Chaudhury et al., 2003; Roopenian and Akilesh, 2007; Akilesh et al., 2008). Likewise, the amount of C[ABD]SL[Eu] was lower than the non-ABD-containing control in the liver. Since proteins entering the liver are eliminated by specific receptors and unspecific phagocytic uptake (Solá and Griebenow, 2011), FcRn recycling is also here a likely explanation for the difference in deposition between the construct with and without ABD. While the difference in kidney deposition between C[ABD]SL[Eu] and the control became apparent already at the first investigated time point (144 h), it was surprising that the highest concentrations of both proteins were reached only after 216 h. This delay in clearance may be explained by the same reasons as for the unexpectedly long serum circulation half-life of the CSL[Eu], i.e., charge and other physicochemical properties.

Despite the potential benefits of half-life extension strategies regarding the *in vivo* efficacy of endolysin-based compounds, it should not be forgotten that any modification of these enzymes, be it through genetic engineering or biochemical approaches, can have a detrimental effect on their antimicrobial activity, which may outweigh the positive effect of an extended half-life on the overall efficacy. This may be due to reduced diffusion, steric hindrance, lower flexibility between individual domains or unpredictable changes affecting the folding of the molecule. Reduced enzymatic and antimicrobial activity was also observed for the C[ABD]S construct compared to the control in this study. The best way to improve the enzymatic activity is probably to work further in detail on the positioning of the ABD to avoid steric hindrance and allow for accurate flexibility between domains. However, it is encouraging to find that the ABD-containing construct was still very potent and reduced *S. aureus* by 3-logs in human serum within 60 min at the tested concentrations (compared to 4 logs for the control). Given this residual activity, our strategy compares favorably with other half-life extension approaches that lead to complete inactivation of the PGH (Walsh et al., 2003; Resch et al., 2011b). A strategy that likely would improve treatment, but possibly complicate the regulatory process, is to administer a mix of both highly active wild type endolysin to get high initial activity and the half-life extended variant to clear any persistent bacteria.

## Conclusion

In conclusion, we have shown that fusion of an endolysin to the ABD represents a promising strategy to extend the serum circulation half-life of a modular PGH, while retaining a considerable level of antimicrobial activity.

## Data Availability Statement

All datasets generated for this study are included in the manuscript and the Supplementary Files.

## Ethics Statement

This study was carried out in accordance with the recommendations of the Guide for the Care and Use of Laboratory Animals of the National Institutes of Health. The protocol ZH251/14 was approved by the Institutional Animal Care and Use Committee of the University of Zurich.

## Author Contributions

JS, MS, AS, YS, and AS conceived and designed the experiments. JS, NK, and AS performed the experiments. JS, MS, NK, YS, and AS analyzed the data. ML and AZ contributed reagents, materials, and analysis tools. JS, MS, YS, and AS wrote the manuscript. JS, ML, and AZ provided funding.

## Conflict of Interest Statement

ML is an advisor for Micreos, a company producing phage-based antimicrobials. The remaining authors declare no conflict of interest. The funders had no role in study design, data collection, data interpretation, or the decision to submit the work for publication.

## References

[B1] Akilesh S. Huber T. B. Wu H. Wang G. Hartleben B. Kopp J. B. (2008). Podocytes use FcRn to clear IgG from the glomerular basement membrane. *Proc. Natl. Acad. Sci. U.S.A.* 105 967–972. 10.1073/pnas.0711515105 18198272 PMC2242706

[B2] Bateman A. Rawlings N. (2003). The CHAP domain: a large family of amidases including GSP amidase and peptidoglycan hydrolases. *Trends Biochem. Sci.* 28 230–234. 10.1016/S0968-0004(03)00062-68 12765834

[B3] Becker S. C. Dong S. Baker J. R. Foster-Frey J. Pritchard D. G. Donovan D. M. (2009). LysK CHAP endopeptidase domain is required for lysis of live staphylococcal cells. *FEMS Microbiol. Lett.* 294 52–60. 10.1111/j.1574-6968.2009.01541.x 19493008

[B4] Bischoff M. Dunman P. Kormanec J. Macapagal D. Murphy E. Mounts W. (2004). Microarray-based analysis of the *Staphylococcus aureus* B regulon. *Society* 186 4085–4099. 10.1128/JB.186.13.4085 PMC421609 15205410

[B5] Bryant P. Ploegh H. (2004). Class II MHC peptide loading by the professionals. *Curr. Opin. Immunol.* 16 96–102. 10.1016/j.coi.2003.11.011 14734116

[B6] Chambers H. F. Deleo F. R. (2010). Waves of resistance: *Staphylococcus aureus* in the antibiotic era. *Nat. Rev. Microbiol.* 7 629–641. 10.1038/nrmicro2200.Waves 19680247 PMC2871281

[B7] Chaudhury C. Mehnaz S. Robinson J. M. Hayton W. L. Pearl D. K. Roopenian D. C. (2003). The major histocompatibility complex-related Fc receptor for IgG (FcRn) binds albumin and prolongs its lifespan. *J. Exp. Med.* 197 315–322. 10.1084/jem.20021829 12566415 PMC2193842

[B8] Daniel A. Euler C. Collin M. Chahales P. Gorelick K. J. Fischetti V. A. (2010). Synergism between a novel chimeric lysin and oxacillin protects against infection by methicillin-resistant *Staphylococcus aureus*. *Antimicrob. Agents Chemother.* 54 1603–1612. 10.1128/AAC.01625-1629 20086153 PMC2849374

[B9] Doweiko J. Nompleggi D. (1991). Role of albumin in human physiology and pathophysiology. *J. Parenter. Enter. Nutr.* 15 207–211. 10.1177/0148607191015002207 2051560

[B10] Fenton M. Casey P. G. Hill C. Gahan C. G. M. Ross R. P. Mcauliffe O. (2010). The truncated phage lysin CHAPk eliminates *Staphylococcus aureus* in the nares of mice. *Bioeng. Bugs* 1 404–407. 10.4161/bbug.1.6.13422 21468207 PMC3056090

[B11] Fischetti V. A. (2010). Bacteriophage endolysins: a novel anti-infective to control gram-positive pathogens. *Int. J. Med. Microbiol.* 300 357–362. 10.1016/j.ijmm.2010.04.002 20452280 PMC3666336

[B12] Green M. R. Sambrook J. (2012). *Molecular Cloning: A Laboratory Manual. Fourth. N.Y*. Cold Spring Harbor, NY: Cold Spring Harbor Laboratory Press.

[B13] Gu J. Xu W. Lei L. Huang J. Feng X. Sun C. (2011). LysGH15, a novel bacteriophage lysin, protects a murine bacteremia model efficiently against lethal methicillin-resistant Staphylococcus aureus infection. *J. Clin. Microbiol.* 49 111–117. 10.1128/JCM.01144-1110 21048011 PMC3020447

[B14] Haddad Kashani H. Schmelcher M. Sabzalipoor H. Seyed Hosseini E. Moniri R. (2017). Recombinant endolysins as potential therapeutics against antibiotic-resistant staphylococcus aureus?: current status of research and novel delivery strategies. *Clin. Microbiol. Rev.* 31:e00071-17. 10.1128/CMR.00071-17 29187396 PMC5740972

[B15] Horgan M. O’Flynn G. Garry J. Cooney J. Coffey A. Fitzgerald G. F. (2009). Phage lysin LysK can be truncated to its CHAP domain and retain lytic activity against live antibiotic-resistant staphylococci. *Appl. Environ. Microbiol.* 75 872–874. 10.1128/AEM.01831-1838 19047377 PMC2632115

[B16] Jawa V. Cousens L. P. Awwad M. Wakshull E. Kropshofer H. De Groot A. S. (2013). T-cell dependent immunogenicity of protein therapeutics: preclinical assessment and mitigation. *Clin. Immunol.* 149 534–555. 10.1016/j.clim.2013.09.006 24263283

[B17] Johansson M. U. Frick I.-M. Nilsson H. Kraulis P. J. Hober S. Jonasson P. (2002). Structure, specificity, and mode of interaction for bacterial albumin-binding modules. *J. Biol. Chem.* 277 8114–8120. 10.1074/jbc.M109943200 11751858

[B18] Jonsson A. Dogan J. Herne N. Abrahmsén L. Nygren P.-A. (2008). Engineering of a femtomolar affinity binding protein to human serum albumin. *Protein Eng. Des. Sel.* 21 515–527. 10.1093/protein/gzn028 18499681

[B19] Jun S. Y. Jang I. J. Yoon S. Jang K. Yu K. Cho J. Y. (2017). Pharmacokinetics and tolerance of the phage endolysin-based candidate drug SAL200 after a single intravenous administration among healthy volunteers. *Antimicrob. Agents Chemother.* 61:e02629-16. 10.1128/AAC.02629-2616 28348152 PMC5444177

[B20] Jun S. Y. Jung G. M. Yoon S. J. Oh M. D. Choi Y. J. Lee W. J. (2013). Antibacterial properties of a pre-formulated recombinant phage endolysin, SAL-1. *Int. J. Antimicrob. Agents* 41 156–161. 10.1016/j.ijantimicag.2012.10.011 23276502

[B21] Jun S. Y. Jung G. M. Yoon S. J. Youm S. Y. Han H.-Y. Lee J.-H. (2016). Pharmacokinetics of the phage endolysin-based candidate drug SAL200 in monkeys and its appropriate intravenous dosing period. *Clin. Exp. Pharmacol. Physiol.* 43 1013–1016. 10.1111/1440-1681.12613 27341401

[B22] Knop K. Hoogenboom R. Fischer D. Schubert U. S. (2010). Poly(ethylene glycol) in drug delivery: pros and cons as well as potential alternatives. *Angew. Chem. Int. Ed. Engl.* 49 6288–6308. 10.1002/anie.200902672 20648499

[B23] Kokai-Kun J. F. Chanturiya T. Mond J. J. (2007). Lysostaphin as a treatment for systemic *Staphylococcus aureus* infection in a mouse model. *J. Antimicrob. Chemother.* 60 1051–1059. 10.1093/jac/dkm347 17848374

[B24] Kontermann R. E. (2011). Strategies for extended serum half-life of protein therapeutics. *Curr. Opin. Biotechnol.* 22 868–876. 10.1016/j.copbio.2011.06.012 21862310

[B25] Kristian S. A. Golda T. Ferracin F. Cramton S. E. Neumeister B. Peschel A. (2004). The ability of biofilm formation does not influence virulence of *Staphylococcus aureus* and host response in a mouse tissue cage infection model. *Microb. Pathog.* 36 237–245. 10.1016/j.micpath.2003.12.004 15043859

[B26] Liu W. Xie Y. Ma J. Luo X. Nie P. Zuo Z. (2015). Sequence analysis IBS?: an illustrator for the presentation and visualization of biological sequences. *Bioinformatics* 31 3359–3361. 10.1093/bioinformatics/btv362 26069263 PMC4595897

[B27] Lobo E. D. Hansen R. J. Balthasar J. P. (2004). Antibody pharmacokinetics and pharmacodynamics. *J. Pharm. Sci.* 93 2645–2668. 10.1002/jps.20178 15389672

[B28] Loeffler J. M. Djurkovic S. Fischetti V. A. (2003). Phage lytic enzyme Cpl-1 as a novel antimicrobial for pneumococcal bacteremia. *Infect. Immun.* 71 6199–6204. 10.1128/IAI.71.11.6199-6204.2003 14573637 PMC219578

[B29] Matthews J. E. Stewart M. W. De Boever E. H. Dobbins R. L. Hodge R. J. Walker S. E. (2008). Pharmacodynamics, pharmacokinetics, safety, and tolerability of albiglutide, a long-acting glucagon-like peptide-1 mimetic, in patients with type 2 diabetes. *J. Clin. Endocrinol. Metab.* 93 4810–4817. 10.1210/jc.2008-1518 18812476

[B30] Nygren P. A. Flodby P. Andersson R. Wigzell H. Uhlen M. (1991). *In vivo* stabilization of a human recombinant CD4 derivative by fusion to a serum-albumin-binding receptor. *Vaccines* 91:363.

[B31] O’Flaherty S. Coffey A. Edwards R. Meaney W. Fitzgerald G. F. Ross R. P. (2004). Genome of staphylococcal phage k: a new lineage of myoviridae infecting gram-positive bacteria with a low G+C content. *J. Bacteriol.* 186 2862–2871. 10.1128/JB.186.9.2862-2871.2004 15090528 PMC387793

[B32] O’Flaherty S. Coffey A. Meaney W. Fitzgerald G. F. Ross R. P. (2005). The recombinant phage lysin LysK has a broad spectrum of lytic activity against clinically relevant staphylococci, including methicillin-resistant *Staphylococcus aureus*. *J. Bacteriol.* 187 7161–7164. 10.1128/JB.187.20.7161-7164.2005 16199588 PMC1251611

[B33] Orlova A. Jonsson A. Rosik D. Lundqvist H. Lindborg M. Abrahmsen L. (2013). Site-specific radiometal labeling and improved biodistribution using ABY-027, a novel HER2-targeting affibody molecule-albumin-binding domain fusion protein. *J. Nucl. Med.* 54 961–968. 10.2967/jnumed.112.110700 23528382

[B34] Patel S. S. Benfield P. (1996). Pegaspargase (polyethylene Glycol-L-Asparaginase). *Clin. Immunother.* 5 492–496. 10.1007/BF03259345

[B35] Rashel M. Uchiyama J. Ujihara T. Uehara Y. Kuramoto S. Sugihara S. (2007). Efficient elimination of multidrug-resistant *Staphylococcus aureus* by cloned lysin derived from bacteriophage phi MR11. *J. Infect. Dis.* 196 1237–1247. 10.1086/521305 17955443

[B36] Reichelt P. Schwarz C. Donzeau M. (2006). Single step protocol to purify recombinant proteins with low endotoxin contents. *Protein Expr. Purif.* 46 483–488. 10.1016/j.pep.2005.09.027 16290005

[B37] Resch G. Moreillon P. Fischetti V. A. (2011a). A stable phage lysin (Cpl-1) dimer with increased antipneumococcal activity and decreased plasma clearance. *Int. J. Antimicrob. Agents* 38 516–521. 10.1016/j.ijantimicag.2011.08.009 21982146

[B38] Resch G. Moreillon P. Fischetti V. A. (2011b). PEGylating a bacteriophage endolysin inhibits its bactericidal activity. *AMB Express* 1:29. 10.1186/2191-0855-1-29 21982426 PMC3222324

[B39] Roopenian D. C. Akilesh S. (2007). FcRn: the neonatal Fc receptor comes of age. *Nat. Rev. Immunol.* 7 715–725. 10.1038/nri2155 17703228

[B40] Schmelcher M. Donovan D. M. Loessner M. J. (2012). Bacteriophage endolysins as novel antimicrobials. *Future Microbiol.* 7 1147–1171. 10.2217/fmb.12.97 23030422 PMC3563964

[B41] Schmelcher M. Shen Y. Nelson D. C. Eugster M. R. Eichenseher F. Hanke D. C. (2014). Evolutionarily distinct bacteriophage endolysins featuring conserved peptidoglycan cleavage sites protect mice from MRSA infection. *J. Antimicrob. Chemother.* 70 1453–1465. 10.1093/jac/dku552 25630640 PMC4398471

[B42] Schuch R. Nelson D. Vincent A. F. (1986). A bacteriolytic agent that detects and kills bacillus anthracis. *Exp. Biol. J. Gen. Physiol.* 124 5–13. 10.1038/nature01026 12192412

[B43] Seijsing J. Lindborg M. Höidén-Guthenberg I. Bönisch H. Guneriusson E. Frejd F. Y. (2014). An engineered affibody molecule with pH-dependent binding to FcRn mediates extended circulatory half-life of a fusion protein. *Proc. Natl. Acad. Sci. U.S.A.* 111 17110–17115. 10.1073/pnas.1417717111 25406323 PMC4260588

[B44] Solá R. J. Griebenow K. (2011). Glycosylation of therapeutic proteins: an effective strategy to optimiza efficacy. *BioDrugs* 24 9–21. 10.2165/11530550-000000000-00000.Glycosylation 20055529 PMC2805475

[B45] Spratt B. G. (1994). Resistance to antibiotics mediated by target alterations. *Science* 264 388–393. 10.1126/science.8153626 8153626

[B46] Verbree C. T. Dätwyler S. M. Meile S. Eichenseher F. Donovan D. M. Loessner M. J. (2017). Identification of peptidoglycan hydrolase constructs with synergistic staphylolytic activity in cow’s milk. *Appl. Environ. Microbiol.* 83 1–15. 10.1128/AEM.03445-3416 28159785 PMC5359494

[B47] Veronese F. M. Mero A. (2008). The impact of PEGylation on biological therapies. *BioDrugs* 22 315–329. 10.2165/00063030-200822050-200822054 18778113

[B48] Walsh S. Shah A. Mond J. (2003). Improved pharmacokinetics and reduced antibody reactivity of lysostaphin conjugated to polyethylene glycol. *Antimicrob. Agents Chemother.* 47 554–558. 10.1128/AAC.47.2.554-558.2003 12543658 PMC151727

[B49] Wang W. Wang E. Q. Balthasar J. P. (2008). Monoclonal antibody pharmacokinetics and pharmacodynamics. *Clin. Pharmacol. Ther.* 84 548–558. 10.1038/clpt.2008.170 18784655

[B50] Werle M. Bernkop-Schnürch A. (2006). Strategies to improve plasma half life time of peptide and protein drugs. *Amino Acids* 30 351–367. 10.1007/s00726-005-0289-283 16622600

[B51] Whisstock J. C. James A. M. (1999). SH3 domains in prokaryotes. *Trends Biochem. Sci.* 24 132–133. 10.1016/S0968-0004(99)01366-1363 10322416

