# Supplementary material for: Improved Biodistribution and Extended Serum Half-Life of a Bacteriophage Endolysin by Albumin Binding Domain Fusion
Source: Front Microbiol. 2018 Nov 27;9:2927. doi: 10.3389/fmicb.2018.02927 (PMC6277698; doi:10.3389/fmicb.2018.02927)
Supplement: Supplementary file 1 [file Data_Sheet_1.docx]

Supplementary Material

Improved biodistribution and extended serum half-life of a bacteriophage endolysin by albumin binding domain fusion

Johan Seijsing^*^, Anna M. Sobieraj, Nadia Keller, Yang Shen, Annelies S. Zinkernagel, Martin J. Loessner, and Mathias Schmelcher

*** Correspondence:** Johan Seijsing: [johan.seijsing@su.se](mailto:johan.seijsing@su.se)


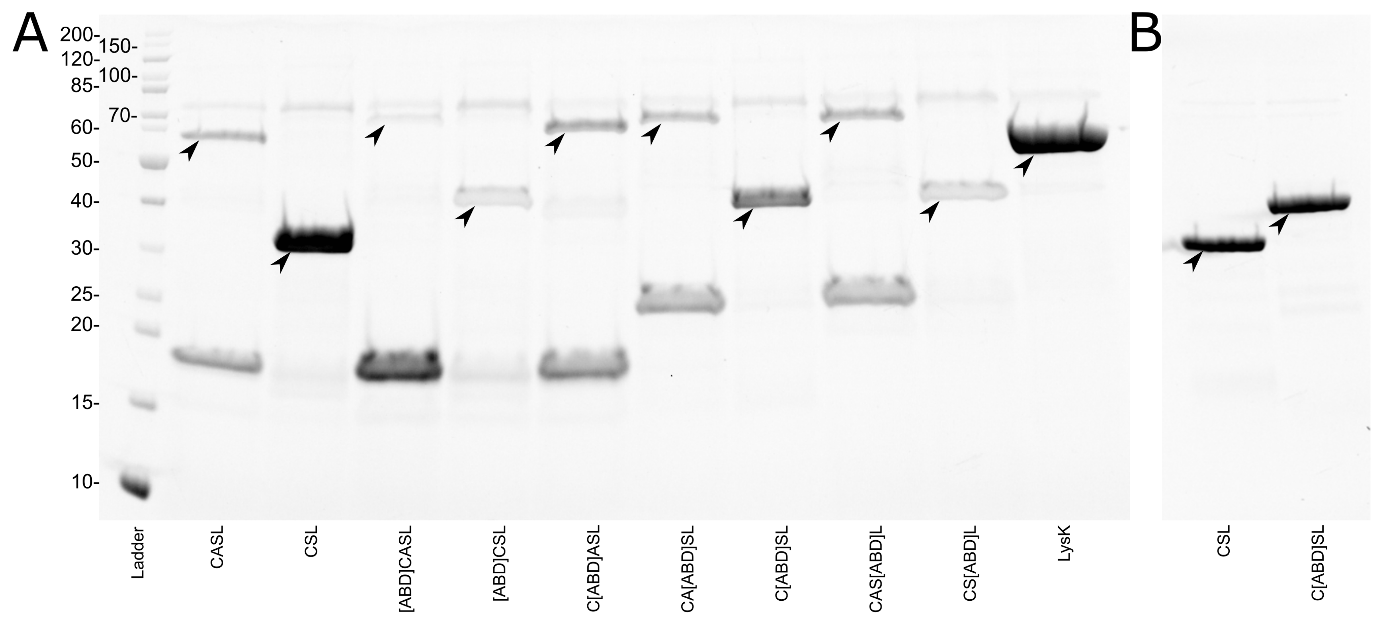


**Supplementary Figure 1.** SDS PAGE of expressed and purified recombinant proteins. Bands of correct size are marked with arrows. (A) Proteins used for in vitro analysis, from left to right: ladder, CASL (59 kDa), CSL (35 kDa), [ABD]CASL (64 kDa), [ABD]CSL (40 kDa), C[ABD]ASL (64 kDa), CA[ABD]SL (64 kDa), C[ABD]SL (40 kDa), CAS[ABD]L (64 kDa), CS[ABD]L (40 kDa) and LysK (56 kDa). (B) Proteins used for in vivo experiments, from left to right: CSL (35 kDa), C[ABD]SL (40 kDa).


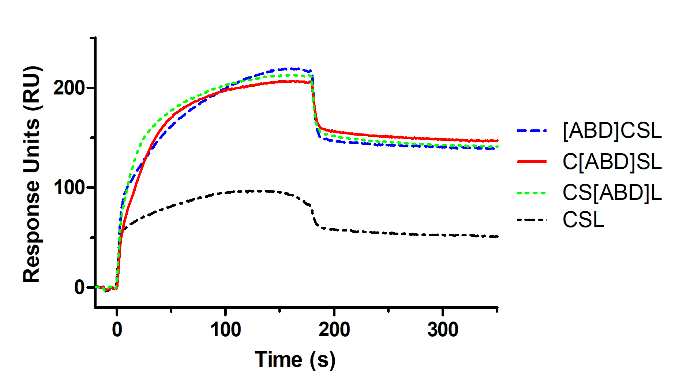


**Supplementary Figure 2.** Surface plasmon resonance sensorgram showing the interaction of fusion proteins containing or lacking ABD with immobilized human serum albumin (HSA).
